# Development of Pressure Sensor Based Wearable Pulse Detection Device for Radial Pulse Monitoring

**DOI:** 10.3390/mi13101699

**Published:** 2022-10-09

**Authors:** Shihang Wang, Zhinan Zhang, Zhijian Chen, Deqing Mei, Yancheng Wang

**Affiliations:** 1 Key Laboratory of Advanced Manufacturing Technology of Zhejiang Province, School of Mechanical Engineering, Zhejiang University, Hangzhou 310027, China; 2 State Key Laboratory of Fluid Power and Mechatronic Systems, School of Mechanical Engineering, Zhejiang University, Hangzhou 310027, China; 3 Donghai Laboratory, Zhoushan 316021, China

**Keywords:** wearable device, pulse detection, pressure sensor, sawtooth protrusions, radial pulse monitoring

## Abstract

Wearable pulse detection devices can be used for daily human healthcare monitoring; however, the relatively poor flexibility and low sensitivity of the pulse detection devices are hindering the scrutiny of pulse information during pulse diagnosis of different pulse positions. This paper developed a novel and wearable pulse detection device based on three flexible pressure sensors using synthetic graphene and silver composites as the pressure sensing material. The structural design of the pulse detection device is firstly presented; the core component of pressure sensors is using the sawtooth protrusions to convert pressure induced by radial pulse vibrations into localized deformation of graphene composites. The fabricated pulse detection device is characterized by high pressure sensing performance, including relatively high sensitivity (8.65% kPa^−1^), broad sensing range (12 kPa), and good dynamic response with a response time of about 100 ms. Then, the pulse detection device is worn on a human wrist to detect the pulses from three pulse positions, namely, ‘Cun’, ‘Guan’, and ‘Chi’, and the results demonstrated the capability of using our device to detect pulse signals. The physical conditions of the subject, such as arterial stiffness index, can be further analyzed through the characteristics of the acquired pulse signals, demonstrating the potential application of using wearable pulse detection devices for human health monitoring.

## 1. Introduction

Several types of deaths caused by diseases are associated with human habits, for instance, deaths caused by cardiovascular disease are increasing in the past decades [1,2]. Continuous monitoring of human health can be used for early diagnosis, better control, and prevention of cardiovascular diseases [3,4]. Thus, health monitoring technologies have attracted people’s attention since they provide the potential to better prevent and treat diseases by monitoring personal health information. Furthermore, with the rapid advancement of smart medicine, IoT technology, and wearable sensing technologies, wearable devices with the ability to monitor the changes of vital signals and used for personalized continuous healthcare have been widely studied [5,6,7].

Pulse signals are regarded as an indispensable indicator and can be used for health monitoring because it contains a wealth of information that can reflect the body’s condition [8,9]. In clinical diagnosis of Chinese traditional medicine, doctors can examine the body’s arterial pulsations by palpation to assess the patient’s health status. The effectiveness, validity, and uniqueness of pulse diagnosis in clinical diagnosis have been reported for thousands of years; there exist several pulse detection devices that can be utilized for primary clinical diagnosis, such as the oximeter, pulse instrument, and cardiovascular function monitor. These devices are usually inconvenient to wear and for use in long-term health monitoring because of their large volume, rigid structures, and poor wearing comfortability. In addition, the clinical treatment and prevention based on Chinese traditional medicine, such as the theory of pulse-taking in three regions and nine divisions, has not been combined with wearable pulse detection devices. The accuracy of pulse diagnosis is highly dependent on the experiences of the doctors, and the accurate perception of pulse signals is vital for the daily monitoring of pulse and the diagnosis of individual health conditions. Currently, several types of wearable pulse detection devices have been proposed, such as volumetric pulse sensors [10,11,12] and pressure pulse sensors [13,14,15]. Volumetric pulse sensors utilize photoplethysmography (PPG) to measure the changes of blood vessel volume to characterize the pulse fluctuations. Yan et al. [16] proposed a GaN integrated optoelectronic device using a ring-shaped structure to achieve pulse detection when using fingertip touching; however, this device was susceptible to the interferences from external light. In contrast, pressure pulse sensors can detect the pressure waves from pulse beating and characterize pulse fluctuations, which corresponds with the ‘Ju’, ‘An’, and ‘Xun’ methods of pulse diagnosis based on Chinese traditional medicine. Lee et al. [17] utilized highly conductive polymers to develop a highly sensitive and stable pressure sensor that can detect the pulse waves during pulse beating. Pressure pulse sensors can accurately detect the pulse waves and they are more compatible with the way the pulse is palpated. Therefore, the development of portable, wearable, and flexible pulse detection devices based on pressure sensors has great significance for accurate pulse diagnosis.

Several studies have been conducted on the development of pulse detection devices based on pressure sensing principles; they can be based on various transduction mechanisms, such as capacitive [18], piezoresistive [19], piezoelectric [20], and triboelectric [21] effects. Among them, sensors based on the piezoresistive sensing principle generally have good flexibility, high sensitivity, and fast response time so that they could be used for the design of pulse detection devices based on pressure sensing. Carbon nanotubes [22], graphene [23], and metal nanowires/nanoparticles [24] have been utilized to fabricate piezoresistive-based sensors and electronic devices. For example, Zhong et al. [25] utilized freeze-drying and post-annealing processes to prepare the wrinkled graphene foam and developed a piezoresistive pressure sensor. It demonstrated good performance in pulse detection. However, the generally complicated materials’ fabrication process limits the fabrication of the device and its further applications. Directly dispersing conductive particles into polymers is an efficient and economical way to fabricate the sensitive conductive elastomers; however, the uniformity of conductive particles dispersing in polymers is a crucial challenge. Apart from sensing material, the structural design of the device also has an important influence on the sensing performance of pulse detection devices. To improve the sensitivity, structural design of pressure-sensing-based pulse detection devices have been conducted, such as using micro-pyramids [26], micro-pillars [27], and nano-cones [28] as the sensing structures. For instance, Bao et al. [29] proposed a tunable method of designing pyramid micro-structured pressure sensors for target sensing requirements, and the fabricated pressure sensors with specialized pyramid microstructures can be applied to the arterial pulse waves detection. Thus, ultrahigh sensitivity of the pulse detection device can be developed with the capability of precisely detecting the pulse beats from radial arteries. Based on the above description, the novel structural design and fabrication process to develop highly sensitive pressure-sensing-based pulse detection devices still need to be investigated and was the goal of this research.

Herein, we present a novel design of wearable pulse detection devices based on pressure sensors for radial pulse monitoring. Typically, the device has three pressure sensors for the detection of three pulse positions, namely the ‘Cun’, ‘Guan’, and ‘Chi’ pulse positions. To achieve both high flexibility and stretchability, graphene and silver nanoflakes were dispersed into elastic silicone rubber to function as the pressure sensing material and stretchable electrodes, respectively. An efficient mixing method was utilized to improve the dispersion uniformity of the nanofillers inside the polymeric matrix. The sawtooth protrusion structure was designed and used as the sensing element to convert external pressure into localized deformation of the graphene composites. Due to the prepared composites and novel structural design, the developed wearable pulse detection device exhibited a relatively high sensitivity of 8.65% kPa^−1^ in a range of 12 kPa for external pressure sensing. Furthermore, this device can be tightly worn on the wrist through a wristband, and radial pulse detection experiments were performed. The detected pulse information, analyzed from waveforms and characterized features of pulse signals, has shown potential to investigate the relationship between pulse signals and health condition of the subject. The obtained results demonstrated the potential of using our proposed wearable pulse detection device for daily human health monitoring.

## 2. Design of Wearable Pulse Detection Device

**Figure 1:**
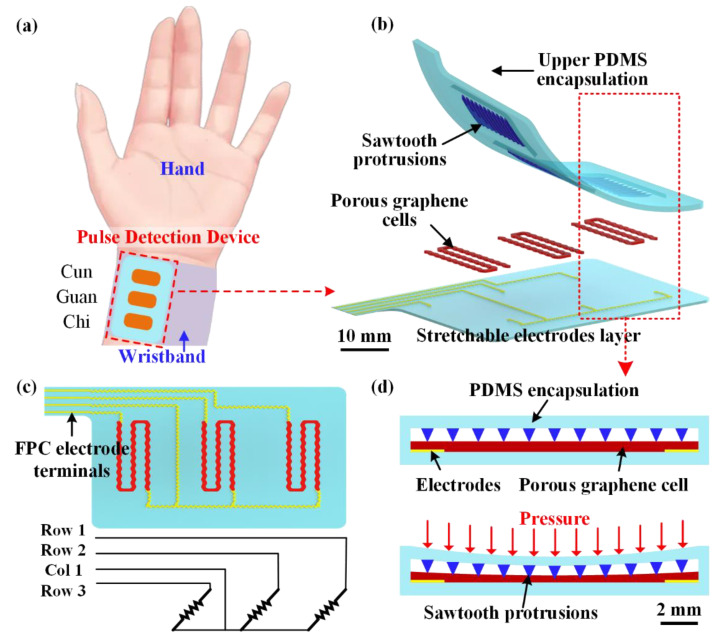
(**a**) Schematic view of wearable pulse detection device for radial pulse monitoring; (**b**) the multilayer structure of pulse detection device; (**c**) electrode connection of porous graphene cells and measurement circuit; (**d**) the pressure-sensing principle of the pulse detection device.

The schematic view of the proposed wearable pulse detection device based on pressure sensors for radial pulse monitoring is illustrated as shown in Figure 1. The wearable pulse detection device has three pressure sensors and it can be mounted onto the wrist via a wristband, as in Figure 1a. The placement locations of the three pressure sensors are designed to be in contact with the ‘Cun’, ‘Guan’, and ‘Chi’ pulse positions by rotating the wristband. As shown in Figure 1b, the pulse detection device mainly consists of three main layers: the bottom stretchable electrode layer, three porous graphene cells, and the upper polydimethylsiloxane (PDMS) encapsulation layer. The serpentine-patterned porous graphene cells are utilized as the pressure-sensitive elements; three sets of sawtooth protrusion structures are designed on the encapsulation layer and they are located above the porous graphene cells. The bottom stretchable electrodes are connected to the ends of the porous graphene cells for electrical transmission and to measure the resistances of the three pressure sensors, as in Figure 1c. As for the electrical circuit, the upper ends of the porous graphene cells are connected to three individual row electrodes, while the lower ends are linked to one public column electrode. The terminals of the stretchable electrodes are designed using a flexible printed circuit (FPC) to connect with peripheral measurement instruments.

The resistance of the circuits is the sum of electrode resistance, porous graphene cell resistance, and contact resistance between the electrode and graphene cell. Whereas the electrode resistance is small and can be neglected, the contact resistance is almost unchanged since invariance of contact area as the external pressure is applied. Thus, the resistance of the circuits mainly depends on the porous graphene cells. To describe the pressure sensing mechanism, the cross-sectional view of sensing elements is illustrated as in Figure 1d. The porous graphene cell is sandwiched by the upper encapsulation and bottom electrode and substrate layer, and there are a set of ten sawtooth protrusions attached onto the upper encapsulation. The sawtooth protrusions are distributed with the same spacing distance and cross the upper surface of the porous graphene cell. As shown in Figure 1d, when pressure is applied to the sensing element, the pressure can be converted into the deformation of graphene cells through the sawtooth protrusions. The position of the porous graphene cells in contact with the sawtooth protrusions plays a role in fulcrums, and then the sawtooth protrusions will stretch the network of porous graphene cells under the action of external pressure and further increase the resistance of the sensing element.

As for material selection, the wearable pulse detection device will be fabricated using fully flexible and stretchable materials. The upper encapsulation is made of PDMS through mold casting, wherein the PDMS for sawtooth protrusions has high rigidness for its different content of curing agent. The substrate of the electrode layer was prepared through PDMS coating. The porous graphene cells and stretchable electrodes have been fabricated by dispersing azodicarbonamide (AC), graphene nanoplates (GNP), and silver (Ag) nanoflakes into silicone rubber (SR) to obtain porous sensing materials (p-GNP-SR) with high sensitivity and electrode materials with high conductivity (Ag-SR), respectively. Thus, we can see that there are no rigid metallic components in the device, and the flexibility of our proposed pulse detection device could be enhanced.

## 3. Experimental Setup and Procedure

### 3.1. Material Preparation and Device Fabrication

To prepare the p-GNP-SR and Ag-SR composites, the GNP (diameter < 10 μm) and Ag were purchased from The Sixth Element Materials Technology Co., Ltd., Changzhou, China and XFNANO Materials Tech Co., Ltd., Nanjing, China, respectively. The silicone rubber (SR, GD401, Zhonghao Chenguang Research Institute of Chemical Industry, Zigong, China) is utilized as the polymeric matrix of the composites. It is a single component rubber that can be cured under heating and no other curing agents are required. The azodicarbonamide (AC, LK-8000, Jia Shi chemical industry Co., Ltd., Jiangmen, China) was selected as the foaming agent. The zinc-oxide (ZnO, Xianfeng Nanomaterial Technology Co., Ltd., Nanjing, China.) was used as the catalyst for the decomposition of the AC foaming agent.

The SR, AC foaming agent, and GNPs were added into a testing tube with mass ratios of 44.8%, 6.7%, and 2.2%, respectively. Then, ZnO powder with a mass ratio of 1.3% as the catalyst for the AC foaming agent, and PPMS (Weng Jiang Reagent Co., Ltd., Guangdong, China) with a mass ratio of 44.8% as the solvent, were added into the mixture. The above mixture was thoroughly blended in a planetary mixer (AR100, Thinky Corporation, Chengdu, China) for 3 min, and the mixture was further dispersed using ultrasonic stirring (FS-300N, Shanghai Sonxi Ultrasonic Ins., Shanghai, China) for 10 min. Thus, the homogeneous and pasty uncured composites were acquired, and the p-GNP-SR composites could be obtained by heating the uncured composites. The principle of the foaming method to fabricate p-GNP-SR composites has been studied and the porous microstructures are shown in our previous work [30]. As for Ag-SR composites, the Ag and SR were mixed with mass ratios of 2.5:1, and the tetrahydrofuran (THF, provided by Sinopharm Chemical Reagent Co., Ltd., Shanghai, China) with a mass ratio of 1 was added to adjust the viscosity of the mixture and enhance the dispersion uniformity of Ag. Then, the mixture was blended in a planetary mixer for 3 min to obtain the Ag-SR composites. In addition, the PDMS used for sawtooth protrusions had a mass ratio of 5:1 with a curing agent, whereas the ratio of the other PDMS and curing agent was 10:1 for encapsulation and substrate layers.

**Figure 2:**
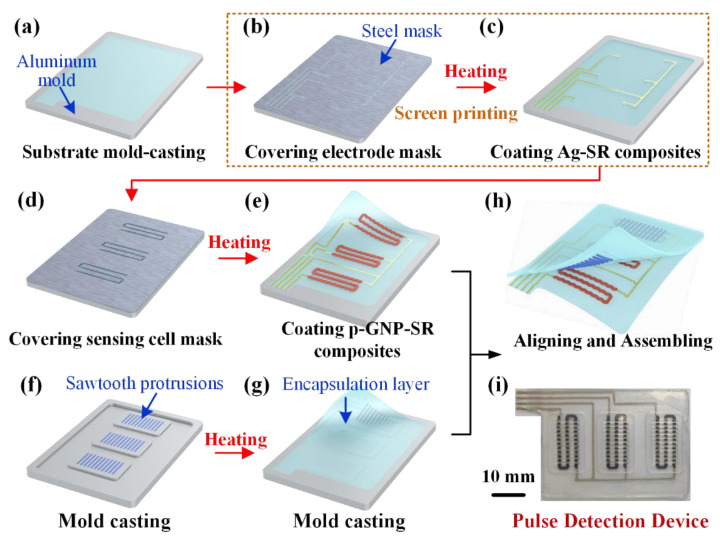
Fabrication process of the wearable pulse detection device: (**a**–**e**) screen-printing of porous graphene cells and stretchable electrodes; (**f**–**h**) mold-casting of the upper encapsulation layer and assembly of the pulse detection device; (**i**) image of the final fabricated device.

The fabrication process to make the wearable pulse detection device can be illustrated as shown in Figure 2. Generally, the fabrication process has four steps.

Step 1: The substrate of stretchable electrode layer was fabricated by using a mold-casting procedure. As in Figure 2a, the prepared uncured PDMS was first poured into a customized aluminum mold. After being degassed in a vacuum chamber, the mold was heated at 80 °C for 2 h to cure the PDMS. Then, the screen-printing method is used to print the electrode pattern onto the PDMS substrate, as shown in Figure 2b,c. A steel mask with stretchable electrode patterned hollows was covered onto the PDMS substrate, and the prepared Ag-SR composites were coated onto the mask. After removal of the mask, the stretchable electrodes on the PDSM substrate were cured under 80 °C for 1 h.

Step 2: The porous graphene cells were fabricated through the same method to screen-print the p-GNP-SR composites onto the fabricated electrode layer, as shown in Figure 2d,e. Then, the PDMS substrate with porous graphene cells and stretchable electrodes were peeled off from the molds.

Step 3: The upper encapsulation with the sawtooth protrusions was fabricated by a typical mold-casting procedure. As in Figure 2f, the aluminum mold with patterned grooves was first customized and the uncured PDMS with high rigidness was poured into the grooves of sawtooth protrusions. After being degassed in a vacuum chamber, the mold was heated at 80 °C for 2 h to cure the PDMS. Then, the other part of upper encapsulation was fabricated using the same procedure, as shown in Figure 2g. Finally, the upper encapsulation was peeled off from the mold.

Step 4: To assemble the pulse detection device, the peripheral edges of upper and bottom layers were coated with a thin layer of uncured PDMS. Then, the upper and bottom layers were pasted to each other after position alignment, and the pulse detection device was further cured for another 2 h to be fully assembled. Due to the identical material utilization of the upper and bottom layers, the PDMS thin layer was able to generate a strong and reliable interfacial bonding between the two layers, so the assembled pulse detection device can be obtained.

The final fabricated wearable pulse detection device is shown in Figure 2i. The black porous graphene cell has been properly connected with the gray electrodes, and the sawtooth protrusions on the upper encapsulation can also be observed, thus demonstrating a good fabrication quality of the pulse detection device.

### 3.2. Characterization of Wearable Pulse Detection Device

**Figure 3:**
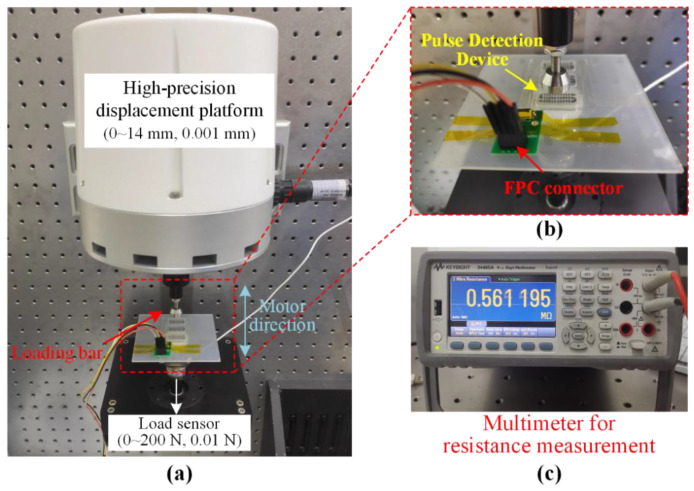
Experimental setup for the characterization of the pulse detection device: (**a**) the performance testing platform setup for the pulse sensor; (**b**) enlarged view of the device mounting and characterization. (**c**) Numerical multimeter for the pressure sensor’s resistance measurement.

The electromechanical properties of the prepared p-GNP-SR and Ag-SR composites have been characterized in our previous work [30,31]. To investigate the sensing performance of the developed pulse detection device, a testing platform (TA Instrument, New Castle, PA, USA) was used. As shown in Figure 3a,b, the pulse detection device was mounted on an acrylic board that was attached to a commercial force sensor with a resolution of 0.01 N and measurement range of 0–200 N. An aluminum loading bar with a diameter of 16 mm was fixed on a high-precision displacement platform to apply incremental pressures to the sensor. The pulse detection device was connected to an FPC connector for resistance measurement using the multimeter (34465A, Keysight, Beijing, China), as shown in Figure 3c.

To intuitively study the deformation of the pressure sensor in the pulse detection device, the pressure sensor was cut apart from the center along the direction perpendicular to the sawtooth protrusions. The cross-sectional surface was observed by a laser confocal microscope (OLS 4100, Olympus, Beijing, China) with a magnification of ×10. For the characterization tests, the loading bar was controlled by the displacement platform, then linearly varying displacement loading on the sensing elements was realized for the characterization of the pulse detection device. The exerted force values applied to the device were measured by the commercial load sensor.

### 3.3. Radial Pulse Monitoring Experimental Procedure

The fabricated pulse detection device was worn on the human wrist with the help of a wearable wristband to carry out the radial pulse monitoring experiments. Three pressure sensors of the pulse detection devices were labeled as ‘Cun’, ‘Guan’, and ‘Chi’ based on the pulse positions to distinguish the sensing units. The device on the wristband was connected to an FPC connector and further linked to the multimeter (34465A, Keysight, Beijing, China). In the pulse detection experiments, the subject was 22 years old with a height of 1.73 m. The pulse detection with a sampling time of 27 s and a frequency of about 14.3 Hz was conducted while the subject sat still. The pulse waves of ‘Cun’, ‘Guan’, and ‘Chi’ pulse positions were detected with the developed device, and the resistance of three pressure sensors in the pulse detection device was measured by the multimeter. Because of the individual differences in pulse intensity, the ratio and time interval of each wave peak within a single pulse wave cycle and the heart rate were the main features in the waveform analysis of pulse waves. So, the collected pulse signals were normalized. In addition, two low-pass filters of order 70 with a cut-off frequency of 0.3 Hz and 4 Hz were utilized to eliminate the low-frequency signal generated by the manual activity and noises.

## 4. Results and Discussion

### 4.1. Characterization Results of Wearable Pulse Detection Device

**Figure 4:**
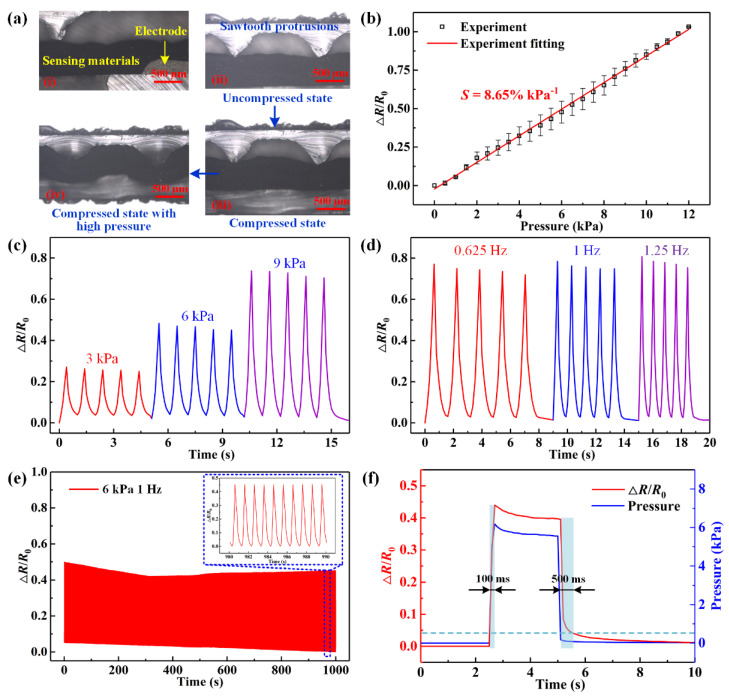
Characterization results of the wearable pulse detection device: (**a**) optical images of cross-section view of the device when examined using a laser confocal microscope; (**b**) pressure sensing sensitivity of the pulse detection device; (**c**–**e**) cyclic tests under different loading pressures, different frequencies, and multiple cycles, respectively; (**f**) response of the device under steady pressure load.

The cross-sectional images of the pulse detection device were examined using the laser confocal microscope and the results are shown in Figure 4a. The images (i) and (ii) are the cross-sectional view of the sensing unit without compression. The porous graphene cells in contact with the sawtooth protrusions can be clearly seen in both images; the porous graphene cell, electrodes, and substrate of the electrode layer were bound tightly with each other. The images (iii) and (iv) show the deformation of the sensor unit under the compressed state with external pressures of 6 kPa and 30 kPa, respectively. The upper sawtooth protrusions squeezed the graphene cells and reduced its sectional area of contact position, which changes the conductive path inside the sensing material and in turn leads to the resistance changes of the sensing elements.

The pressure sensing performance of the pulse detection device was tested and the results are shown in Figure 4b–f. As shown in Figure 4b, as the applied pressure increased, the generated Δ*R*/*R*_0_ of the sensor gradually increased and behaved with good linearity in a range of 0–12 kPa. The sensitivity of pressure sensing can be calculated as *S* = (Δ*R*/*R*_0_)/*P*, where *P* is the applied pressure. In this study, one sensing element was tested three times and its sensitivity can be calculated as *S* = 8.65% kPa^−1^ with R^2^ = 0.9977, according to the experimental data. Secondly, repeated cyclic tests were implemented to the device to verify its good repeatability. As in Figure 4c, a multiple cycle test with incremental pressure loading was conducted. Pressures of 3 kPa, 6 kPa, and 9 kPa were respectively applied to the pulse detection device four times. The Δ*R*/*R*_0_ of each sensor changed regularly and behaved with the same varying tendency versus the applied pressures. The values of Δ*R*/*R*_0_ under 3 kPa, 6 kPa, and 9 kPa were about 0.25, 0.48, and 0.73, respectively, and approximately consistent with the calibration results. It can be noted that there was a small rise in the initial values under different pressures, and a slight drop in the peak values under the same pressure This observation can be attributed to the viscoelasticity and hysteresis of the p-GNP-SR composites; a similar phenomenon has been reported in some related studies [32,33]. Then, the cyclic test with incremental loading frequencies was conducted. As shown in Figure 4d, pressure loadings of 9 kPa at different frequencies of 0.625 Hz, 1 Hz, and 1.25 Hz were applied; the corresponding Δ*R*/*R*_0_ of the pressure sensor closely followed these variations and exhibited fast response, and the corresponding Δ*R*/*R*_0_ peak maintained almost the same at about 0.73. This behavior demonstrates that the device possessed a good dynamic response.

Furthermore, a multiple cycle test was implemented under 6 kPa and the corresponding Δ*R*/*R*_0_ of the pressure sensor was plotted as shown in Figure 4e. The element showed a regularly varied Δ*R*/*R*_0_, although the initial and peak values of the Δ*R*/*R*_0_ gradually declined. However, the relative change of Δ*R*/*R*_0_ remained approximately consistent after 300 time cycles and the drop of initial and peak values was not significant, indicating that the device has a relative long service life. In addition, experiments of the dynamic characteristics of the device were also conducted, and the results are shown in Figure 4f. We can see that the related Δ*R*/*R*_0_ had fast response with external pressure loading and unloading. The value of related Δ*R*/*R*_0_ reached 0.45 as the pressure applied on the element was 6 kPa, and the loading pressure and corresponding Δ*R*/*R*_0_ have a small drop after them reaching the peak. This phenomenon is caused by the viscoelasticity of the p-GNP-SR composites. During the unloading stage, the external pressure decreased fast, while the corresponding Δ*R*/*R*_0_ dropped rapidly at first and then slowly, indicating this device has hysteresis in unloading. The response time of the pressure sensor was about 100 ms, while the recovery took over 500 ms. Overall, the fabricated wearable pulse detection device performed with relatively high sensitivity, good dynamic response, and considerable repeatability.

A comparison on the pressure sensitivity, sensing range, and response time of our pulse detection device and those in some other related research was conducted and is shown in Table 1. Our developed device performed with a relatively high sensitivity (8.65% kPa^−1^) in the pressure sensing range of 0–12 kPa and exhibits fast response time (~100 ms) and recovery time (~500 ms). In overall consideration, the fabricated wearable pulse device performed with relatively high sensitivity, adequate sensing range, good dynamic response, and considerable repeatability. Thus, the pulse detection device would have good potential in radial pulse monitoring when worn on the wrist.

### 4.2. Radial Pulse Monitoring Experimental Results

**Figure 5:**
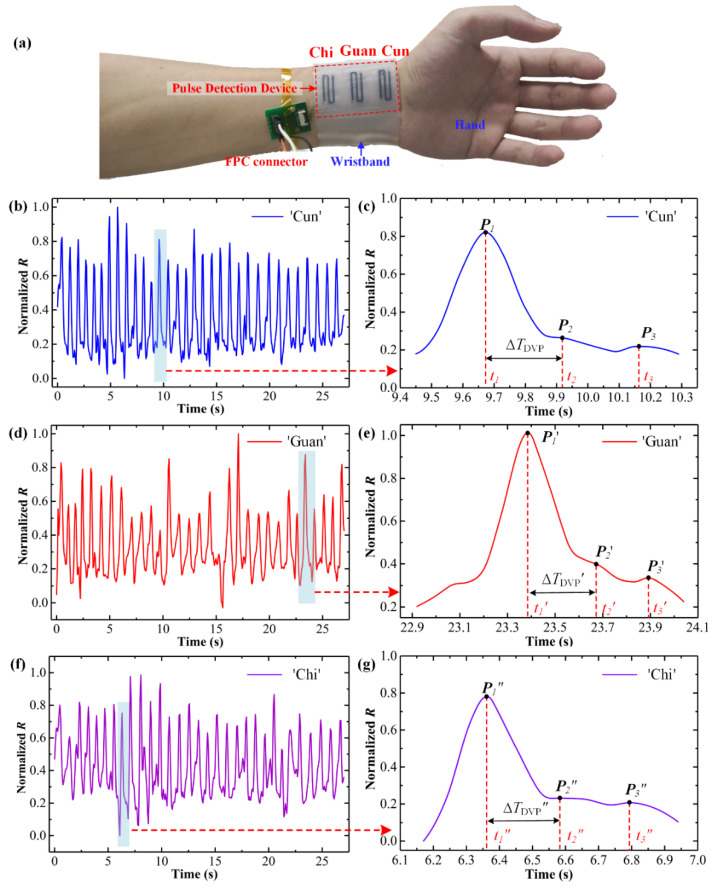
Radial pulse monitoring results: (**a**) pulse detection device worn on the wrist; (**b**–**g**) pulse signals and their enlarge views of ‘Cun’, ‘Guan’, and ’Chi’ pulse positions.

After performance characterization, the fabricated pulse detection device was worn on a human wrist with the help of a wearable wristband to monitor the radial pulse of pulse positions. As shown in Figure 5, a young man was selected as the subject in this section. The pulse detection experimental procedure mainly contained these steps: still sitting of the subject, radial pulse detection of pulse positions, collected data normalization, and signals filtering. The radial pulse detection experiments started after the subject sat still for several minutes. The Δ*R*/*R*_0_ of each pressure sensor in the pulse detection device was measured by the multimeter in the radial pulse detection experiments. The collected pulse data from the three pulse positions were normalized and filtered, then plotted in Figure 5b–g.

As for the pulse signal of the ‘Cun’ position shown in Figure 5b, the normalized *R* (N*R*) rises and falls regularly as the pulse vibrates. The human subject’s pulse fluctuated 32 times in 27 s, and his heart rate is calculated as about 71.1 bpm. This heart rate is normal and healthy for the sedentary subject, which demonstrates the accuracy of the device for pulse detection. The difference of peaks in various pulse cycles was caused by the inevitable hand motions while the device was worn. Figure 5c exhibits a single cardiac cycle between 9.45 s and 10.30 s of the ‘Cun’ position detection signal. The signal rises rapidly and reaches the first peak *P*_1_ at *t*_1_ = 9.67 s. Then, the curve gradually decreases and rises again at *t*_2_ = 9.92 s to reach the second peak *P*_2_. After that, the third peak *P*_3_ of the signal appears at *t*_3_ = 10.16 s. Whereas *P*_1_ is the sum of the incident wave and reflected wave from the hand, *P*_2_ is the peak of the reflected wave from the lower body minus the end-diastolic pressure, and *P*_3_ is called diastolic waves. So, the normalized *R P*_1_ (N*R*(*P*_1_)) is generally larger than N*R*(*P*_2_) and N*R*(*P*_3_). To reflect the characteristics of the pulse signal and supply the diagnostic index of arteriosclerosis for artery stiffness, the time difference (Δ*T*_DVP_) between *P*_1_ and *P*_2_ was defined as the digital volume pulse and calculated as 250 ms in a single cardiac cycle. The radial artery augmentation index (*AI*_r_), defined as *AI*_r_ = N*R*(*P*_2_)/N*R*(*P*_1_), is calculated as 0.32. In addition, arterial stiffness index (*SI*) can be calculated as *SI* = *h* (in m)/Δ*T*_DVP_ (in s) = 6.92, reflecting pulse wave velocity to ensure regional arterial stiffness [44]. According to Traditional Chinese Medicine (TCM), the *P*_2_ of radial pulse waves for old or sick people tends to disappear or approach the *P*_1_ because of high artery stiffness and poor elasticity of blood vessels. This phenomenon causes high *AI*_r_, short Δ*T*_DVP_, and large *SI*. Compared with related research [7], the subject in this study has low *AI*_r_, long Δ*T*_DVP_, and small *SI*, and the values of them were 0.32, 250 ms, and 5.24, respectively. This result corresponds to the young age of the subject and indicates he is healthy. Therefore, the proposed pulse detection device can provide crucial information for palpation and has the potential for development in intelligent health monitoring.

For the pulse signals of the ‘Guan’ position shown in Figure 5d and its single cardiac cycle between 22.92 s and 24.44 s shown in Figure 5e, the pulse signal fluctuated 30 times in 27 s and the heart rate is calculated to be about 67.8 bpm. The first peak, *P*_1′_, appeared at the middle of the cardiac cycle *t*_1′_ = 23.38 s. The detected pulse signal reaches the other peaks, *P*_2′_ and *P*_3′_, at *t*_2′_ = 23.66 s and *t*_3′_ = 23.89 s. The time differences between peaks occupied fewer percentages of a cardiac cycle than that of the ‘Cun’ pulse signal. Given that the time difference of peaks and the radio of N*R* (peaks) changed, the radial artery augmentation index (*AI*_r_’) and arterial stiffness index (*SI*’) were calculated to be 0.25 and 6.17, respectively. In addition, for the pulse signals of ‘Chi’ position shown in Figure 5f and its single cardiac cycle between 6.17 s and 6.95 s shown in Figure 5g, the heart rate is calculated to be about 68.9 bpm. The detected pulse signal reaches peaks *P*_1_”, *P*_2_”, and *P*_3_” at *t*_1_” = 6.36 s, *t*_2_” = 6.59 s, and *t*_3_” = 6.79 s. The radial artery augmentation index (*AI*_r_”) and arterial stiffness index (*SI*”) were 0.30 and 7.52, respectively. The diagnosis indexes *AI*_r_, Δ*T*_DVP_, and *SI* calculated from the pulse waves of ‘Guan’ and ‘Chi’ positions were close to the diagnosis indexes from ‘Cun’ pulse waves, indicating the subject had good health. We can see that the waveforms of ‘Cun’, ‘Guan’, and ‘Chi’ have differences. This phenomenon occurs because the pulse waves of different positions reflect different internal organs’ conditions, according to TCM. Meanwhile, the difference in pulse waves can be utilized to analyze the health of the whole body based on the pulse diagnosis principle of three parts and nine pentads. The waveform disparity per cycle was large, because this device was too wide to fit well to the wrist surface and the inevitable hand motions caused non-pulse generated pressure changes. Despite this, the developed pulse detection device can obtain the pulse signals and display scrutiny features in the signals. Thus, it would have great potential to monitor human health for daily healthcare and drive the development of digital pulse diagnosis.

## 5. Conclusions

In summary, we developed a novel wearable pulse detection device based on highly sensitive pressure sensors for radial pulse monitoring. The pulse detection device was designed and fabricated with elastic polymers; the graphene and silver nanoflakes were dispersed into the silicone rubber to serve as the pressure sensing material and stretchable electrodes, respectively. The sawtooth protrusion structure was designed to convert external pressure into local deformation of the graphene composites and leads to resistance change for signal detection. The fabricated pulse detection device exhibited relatively high sensitivity of 8.65% kPa^−1^ in the range of 12 kPa, and the device can be successfully integrated on a wristband to detect the pulse wave of ‘Cun’, ‘Guan’, and ‘Chi’ pulse positions. The pulse signals during the whole detecting process could be detected by the pulse sensors. Through analysis of the pulse signals of the three pulse positions in feature points and waveform, the heart rate, radial artery augmentation index, and arterial stiffness index were investigated. The heart rate, radial artery augmentation index, and arterial stiffness index from ‘Cun’ pulse waves were calculated as 71.1 bpm, 0.32, and 6.92, respectively. These results demonstrated that the subject had a normal artery stiffness and was healthy.

The relation between the pulse signals of the three pulse positions and physical health condition, such as blood pressure, can be thoroughly studied through the pulse detection device. In addition, the positions of ‘Cun’, ‘Guan’, and ‘Chi’ vary for different people. In future work, we will optimize the structure of the device and wristband to be adjustable and match with various pulse positions of different people. The signal analysis and processing techniques with the combination of pulse theory of Chinese traditional medicine to conduct more pulse detection experiments for different subjects will be investigated.

## Figures and Tables

**Table 1:** The comparison of the sensing performance in this paper and some other related works.

Ref.	Materials	Sensing Mechanism	Sensitivity	Sensing Range
[34]	CB/PU sponge	Piezoresistive	6.80% kPa^−1^	0–2.2 kPa
[35]	Si	Piezoresistive	0.05% kPa^−1^	−10–10 Pa
[36]	CCGF	Piezoresistive	0.36 kPa^−1^ 4.60% kPa^−1^	0–2 kPa 2–5 kPa
[37]	Two single-layer graphene	Piezoresistive	−0.24 kPa^−1^ 3.9% kPa^−1^	0.25–0.7 kPa 1–8 kPa
[38]	Graphene/Ag	Piezoresistive	1.6% kPa^−1^	0–40 kPa
[39]	Micro-structured graphene	Piezoresistive	−5.53 kPa^−1^	0.0015–1.4 kPa
[40]	Graphene	Capacitive	0.2% kPa^−1^	0–450 kPa
[41]	PDMS-AgNWs-CPI	Capacitive	1.19 kPa^−1^ 7.70% kPa^−1^	0–3 kPa 3–15 kPa
[42]	MXene/PVDF-TrFE	Capacitive	0.51 kPa^−1^ 1.10% kPa^−1^	0–1 kPa 1–167 kPa
[26]	PDMS-Ti/Au	Capacitive	0.16 kPa^−1^ 0.04 kPa^−1^	0–0.75 kPa 0.75–2.5 kPa
[43]	ITO-Graphene FET-PDMS	Piezoelectric	0.94% kPa^−1^	0–1.4 kPa
This work	p-GNP-SR	Piezoresistive	8.65% kPa^−1^	0–12 kPa

## Data Availability

The data of characterization and pulse detection experimental tests are available upon written request.
